# Identification of targeted therapy options for gastric adenocarcinoma by comprehensive analysis of genomic data

**DOI:** 10.1007/s10120-020-01045-9

**Published:** 2020-02-27

**Authors:** Daniel A. Hescheler, Patrick S. Plum, Thomas Zander, Alexander Quaas, Michael Korenkov, Asmae Gassa, Maximilian Michel, Christiane J. Bruns, Hakan Alakus

**Affiliations:** 1 Department of General, Visceral and Cancer Surgery, University Hospital of Cologne Germany, Cologne, Germany; 2 Gastrointestinal Cancer Group Cologne (GCGC), University Hospital of Cologne Germany, Cologne, Germany; 3 Department of Medicine I, University Hospital of Cologne Germany, Cologne, Germany; 4 Institute of Pathology, University Hospital of Cologne Germany, Cologne, Germany; 5 Department of Cardiothoracic Surgery, University Hospital of Cologne Germany, Cologne, Germany; 6 Institute of Zoology, University of Cologne Germany, Cologne, Germany

**Keywords:** Targeted molecular therapy, New treatment advances, Human genome project, Stomach neoplasms

## Abstract

**Background:**

So far only trastuzumab, pembrolizumab and ramucirumab have been approved by the FDA for targeted therapy in gastric cancer (GC). Here we report on potential targeted therapy options for gastric adenocarcinoma based on a novel analysis of “The Cancer Genome Atlas (TCGA)” database.

**Methods:**

One hundred two FDA-approved targeted cancer drugs were compiled and molecular targets defined. Drugs were considered as potentially effective if targeted genes showed (1) an increase in copy number, (2) gain of function with oncogene activation, (3) specific alterations responsive to approved drugs. Additionally, genetic changes that confer drug resistance and/or sensitivity were evaluated.

**Results:**

Fifty percentage of patients with GC may be treatable with non-GC but FDA-approved targeted cancer therapies. The major drug identified in our in silico study for GC is copanlisib, a PI3K inhibitor. In the TCGA patient database, our genetically based drug response prediction identified more patients with alterations sensitive to copanlisib compared to the already-GC-approved drug trastuzumab (20%, 78 out of 393 patients, vs. trastuzumab: 13%, 52 of 393 patients), which is mainly due to the high incidence of PIK3CA gain of function mutations within mutation hot spots.

**Conclusion:**

Our results demonstrate that various currently FDA-approved drugs might be candidates for targeted therapy of GC. For clinical trials, cancer patients should be selected based on the genomic profile of their tumor.

**Electronic supplementary material:**

The online version of this article (10.1007/s10120-020-01045-9) contains supplementary material, which is available to authorized users.

## Introduction

The incidence of gastric cancer (GC) has been declining during the last decade, but unfortunately it is still the fifth most frequently diagnosed cancer with the third highest mortality rate among cancers [1]. Diagnosis of GC usually occurs at an advanced, incurable stage (stage III–IV) as early stages (stage I–II) do not exhibit clear symptoms [2]. Therefore, better molecular understanding and diagnostic imaging is urgently needed in order to identify early stages of GC which are potentially curable by endoscopic therapy (stage T1aN0M0). Stages higher than T1bNxMx require partial or full stomatic removal with concomitant neoadjuvant and adjuvant therapy.

Recent advances in targeted therapies have led to an improved prognosis in patients with advanced, unresectable GC. One success story is human epidermal growth factor receptor 2 (HER2) encoded by the ERBB2 gene. HER2 overexpression is detectable in 13–23% of all GCs [3], and the addition of the monoclonal antibody against erbB-2 (Her-2/neu), trastuzumab to chemotherapy resulted in a significant advantage of patient survival for HER2 overexpressing GC patients (median OS: 13.8 vs. 11.1 months, hazard radio HR: 0.74; 95% confidence interval: 0.60–0.91, *p* = 0.0046) [4]. The ToGA trial [4] led to FDA approval of trastuzumab in 2010 and is since the standard of care in combination with a fluoropyrimidine—platinum-based chemotherapy in the first-line setting for advanced HER2 positive GC [5]. Other receptors have likewise been targeted in this context. Ramucirumab, a human monoclonal antibody against the vascular endothelial growth factor receptor 2 (VEGFR-2), was introduced in the REGARD trial [6] and showed a marginal but statistically significant effect compared to placebo (median OS: 5.2 vs. 3.8 months; HR 0.776; 95% CI: 0.603–0.998; *p* = 0.047) [6]. The RAINBOW trial [7] studied the combination of ramucirumab with the chemotherapeutic agent paclitaxel and showed a survival advantage of the combination to therapy with paclitaxel alone (median OS: 9.6 vs. 7.4 months; HR 0.807; 95% CI: 0.678–0.962; *p* = 0.017) [7]. Consequently, this combination was approved by the FDA as a second-line therapy in advanced GC. The FDA also recently approved pembrolizumab for patients with recurrent locally advanced or metastatic GC, whose patient’s tumors express PD-L1 and no EGFR or ALK genomic aberrations. This FDA approval is based on the results of the KEYNOTE 059 trial [8], which showed an objective response rate of 60% (95% CI, 38.7–78.9) in combination therapy and 25.8% (95 CI 11.9–44.6) as monotherapy.

The above-mentioned targeted cancer therapies are so far the only ones approved for GC treatment as many others failed over the past few years. This is in contrast to many other cancer types that can nowadays be treated with targeted drugs.

We hypothesize that careful selection of patients is key for successful targeted therapies in patients with GC. Although many basic molecular biological and genomic data are available for GC, these data have not been carefully analyzed in a clinical context and for patient subgroups that might benefit from already-existing targeted therapeutic drugs used in other cancer types. Therefore, existing data need to be analyzed more comprehensively.

One of the most profound sources of genomics in GC is the data from “The Cancer Genome Atlas” (TCGA) published in 2014 [9]. The TCGA provisional study classified GCs based on genomic and molecular biological features from 478 patients with primary gastric adenocarcinoma into 4 subtypes: (a) Epstein-Barr virus positive (EBV 9%), (b) microsatellite instable tumors (MSI 22%), (c) genomically stable tumors (GS 20%) and (d) chromosomally instable tumors (CIN 50%) [9]. This study introduced a novel classification system and was primarily aimed to search for new GC markers. Consequently, these data were only partially linked to potential targeted therapies. Here, we utilized the TCGA dataset in order to identify new targeted therapeutic options in GC.

## Methods

**Fig. 1:**
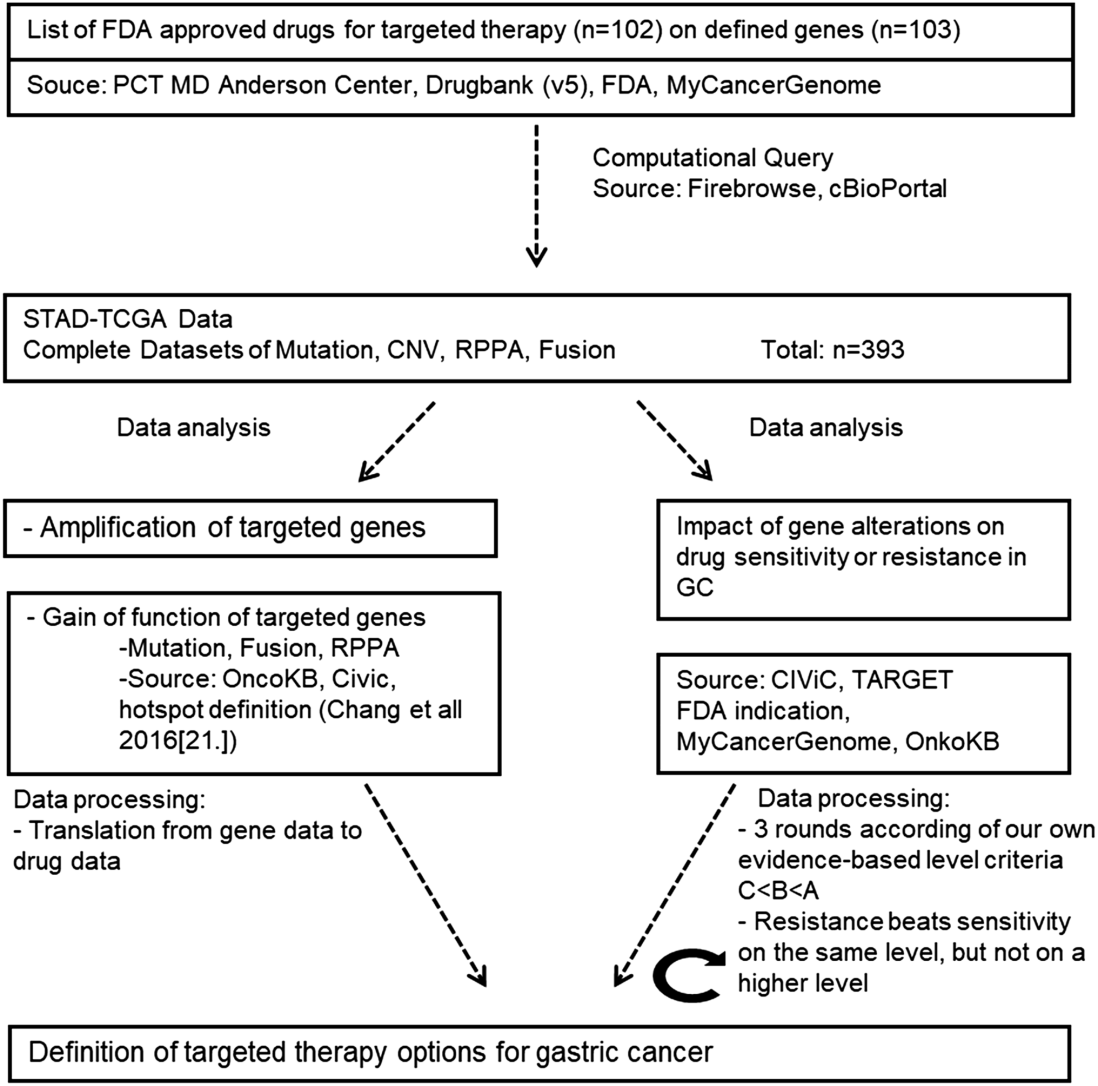
Flowchart of data processing: shown are the major steps of data analysis based on the computational query of drugs for targeted therapy on the respective patient’s data with gastric cancer (GC). Further analysis took into consideration gene amplification and gain of function as well as known alterations of genes to reach a conclusion on target options for GC. Abbreviations: FDA Food and Drug Administration; TCGA The Cancer Genome Atlas [9]; CNV Copy Number Variants based on GISTIC 2.0 algorithm [20]; MAF Mutation Annotation Format based on MutSig 2CV algorithm [19]; RPPA reverse phase protein array based on *Z*-score (overexpression *z* ≥ 2, underexpression *z* ≤ − 2) [18]; gene fusion data were taken out of the supplementary of the STAD-TCGA publication [22]; STAD stomach adenocarcinoma; CiViC Clinical Interpretation of Variance in Cancer database [14]; MyCancerGenome database [11]; DoCM Database of Curated Mutations database [46]; OncoKB Precision Oncology Base databases (drugs actionable variance, all variance with biological effects [16]); PCT MD Anderson Center for Personalized Cancer Therapy [13]; COSMIC Catalogue of Somatic Mutations in Cancer database on drug resistance [47].

The flowchart of our methodological approach and the software pipeline, programmed in visual basic for MS-Excel, is outlined in Fig. 1. In order to find new therapeutic options in GC, first all FDA-approved drugs for any cancer therapy were identified and linked to their respective gene targets, and then, the GC TCGA data were mined for alterations that could be targeted with any of the FDA-approved drugs.

### FDA-approved drugs for targeted cancer therapy and their biological targets

The databases of the National Cancer Institute [10], MyCancerGenome [11] and DrugBank [12] were searched for FDA-approved targeted drugs for the treatment of cancer in 02/2019. Unspecific drugs such as tretinoin or cabazitaxel were excluded (Supplementary Table 1). The candidate list was annotated with the genetic alteration targeted by the drug using the National Cancer Institute [10], MyCancerGenome [11], DrugBank [12] and Anderson Cancer Center [13] databases. Special attention was given as to whether a particular genetic alteration would confer sensitivity or resistance to the targeted therapy. For this, data from MyCancerGenome [11], CiViC [14], TARGET [15] and OncoKB [16] were mined (Supplementary Table 11). Since this resulted in partially overlapping data, we set the following criteria to signify whether a given genetic alteration confers sensitivity or resistance to a given medication with Level *A* > Level *B* > Level *C*:

- *Level A* CiViC level *A* and *B*, FDA indication, MyCancer Genome, OncoKB level 1, 2a, R1
- *Level B* CiViC level *C* and *D*, OncoKB level 3a
- *Level C* Targeted Database all levels, CiViC level *E*, OncoKB level 4

### Genetic alterations in gastric adenocarcinoma

TCGA data are available through the FireBrowse [17] and cBioPortal [18] platforms, as well as supplementary material of the TCGA-STAD publication [9]. These portals allow the user to mine the TCGA data based on recent insight. We specifically utilized (1) mutation data from whole exome sequencing generated by MutSig2CV [19], (2) putative copy number alterations from GISTIC 2.0 [20], (3) protein expression measured by reverse-phase protein array (RPPA) [21], (4) gene fusion information obtained from the supplementary S3.8 “Low-pass structural rearrangements” [22], (5) clinical, pathological information, (6) mononucleotide and dinucleotide marker panel analysis status, as well as (7) TCGA classification and clusters analysis of GC.

We used the FireBrowse [17] and cBioPortal [18] platforms to find the occurrence of genomic and proteomic alterations in all GC patients analyzed in 04/2019. Out of a total of 478 registered patients, we included 393 patients based on a complete dataset of tumor samples with sequencing mutation data as well as gene copy number variation (CNV). We looked for mutation variants, CNVs and protein expression changes in GC tissue samples and found in total 24,538 mutation variants in 1877 genes (Supplementary Table 2), 1902 genes for copy number alterations (Supplementary Table 3) and 192 genes for protein expression changes (Supplementary Table 4) with GC.

We annotated each genetic alteration based on the likelihood for functional impact as follows.

*Gain of function* Gene alterations (i.e., mutation, fusion, amplification) were scored as proposed by OncoKB [16] and Civic [14]. These databases derive a biological effect score from publications. Activating gene alterations were annotated with OncoKB’s “(likely) gain of function,” a Civic clinical significance score of “(likely) pathogenic” or “positive” as well as whether the alteration was in a hotspot as defined by Chang et al. 2016 [23] (Supplementary Table 12).

*Copy number alteration* The data from CBioPortal [18] is annotated with a copy number analysis algorithm (GISTIC 2.0 [20]) which indicates the copy number level per gene: “− 2” deep loose, “− 1” shallow loose, “0” diploid, “1” low-level gain and “2” high-level amplification. We used the threshold of high-level amplification “2” to signify an occurrence of a copy number increase in a given tissue sample.

*Protein expression* Here, cBioPortal [18] reports the relative transcription of an individual gene’s protein expression in tumor tissue with respect to a reference population. We defined as significantly aberrant a *Z*-score more than two standard deviations (SD) higher than the reference population. Protein expression was only available for 192 genes and used primarily for HER2 or estrogen receptor status.

### Drug response prediction: classification of patient tissue into medication sensitive or resistant

We scored whether a given GC patient could have responded to any of the approved drugs. For this, we integrated the data on approved drugs and their targets (Supplementary Table 1) with data on patient tissue samples annotated with the biologically relevant genetic alterations (Supplementary Table 11). We predicted whether a patient might respond to a given drug based on the following criteria:

(i) The gene underlying the FDA-approved drug target shows a copy number increase in the GC tissue of the TCGA study
(ii) The drug targets a gene whose product shows a gain of function in the TCGA tissue
(iii) The drug shows a literature-based effectiveness on a specific alteration found in the TCGA tissue such as indicated in the FDA guidelines.

We processed the data based on our definition of drug sensitive or resistant according to our defined levels of evidence (Level *C* < *B* < *A*, see above). In the case that both a score for “sensitivity” and one for “resistance” occurred in the same level, we scored the gene as conferring “resistance” since a gene conferring resistance to a drug would be highly detrimental to the patient. Where different levels of evidence conflicted, we decided based on the next higher level of evidence (*A* > *B* > *C*). If a final score of “resistance” occurred, the drug was excluded as a potential candidate.

### Subclass and genetically based drug response prediction analysis

Patients from the TCGA databank were grouped into “patients with possible targeted therapy alterations” (Class *A*) and “patients without targeted therapy alterations” (Class B) based on all 295 tumor samples published in the TCGA-STAD 2014 [9] study. Class *A* and Class *B* patients were compared according to the following groups: Molecular Classification of the Asian Cancer Research Group, the molecular Classification of the STAD-TCGA, microsatellite instability, hypermutated, clusters on RNA/miRNA/CNV/methylation, pathology, staging, grading, location, outcome, race as well as gender. The significance of difference in the treatment groups was analyzed by a *χ*^2^ test with a significance level of *p* < 0.05.

We compared between drugs by calculating the confidence interval for the difference between two proportions of potentially responding patient using the IMSIE web calculator [24]. Specifically, we compared each group to the proportion of patients predicted to respond to the FDA-approved drug trastuzumab.

## Results

### FDA-approved drugs for targeted cancer therapy and gene list

In order to detect possible new drugs for GC treatment, we identified 102 FDA-approved drugs for targeted therapy of any cancer type. We linked these drugs to 103 genes which encode the potential sites of binding and action (Supplementary Table 1). Since a given gene alteration can either confer sensitivity or resistance to a drug, we also identified the type of alteration required for therapeutic action. This list of genes was then subjected to a detailed analysis for potential relevance in GC.

### Mutation variants

The list of genes for targeted therapy was analyzed for hot spots of mutation variants as well as mutations known to be responsive to FDA-approved drugs from the literature (Supplementary Table 6). The following genes were found to be hot spots for mutations in the patient datasets analyzed:

- RNF43 at position 659 (34 of 393 patients), which has been reported as a critical negative feedback regulator of the Wnt pathway and results in loss of function of a ubiquitin E3 ligase [25];
- TP53 at position 273 (19 of 393 patients), which is one of the most frequent mutations in several cancer types [26].
- Phosphatidylinositol-4,5-biphosphate 3-kinase catalytic subunit alpha (PIK3CA) at positions 545 (E545K, *n* = 13/300), 1047 (H1047R, *n* = 14/393) and 542 (E542K, *n* = 7/393) which for example plays an important role in drug resistance to EGFR TKI [27].

### Copy number variation

**Fig. 2:**
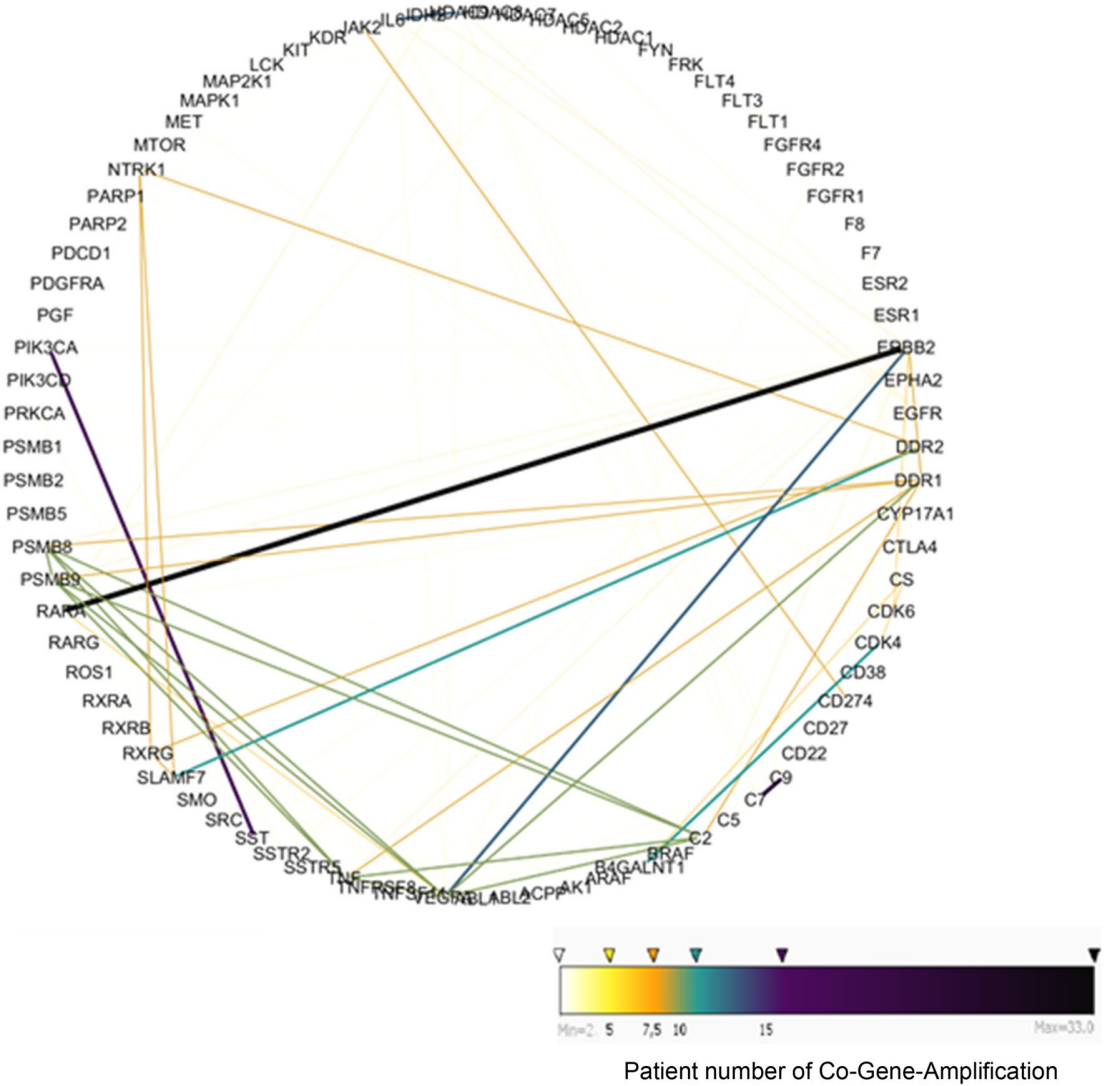
A network of gene amplification observed in the TCGA patients. Lines signify co-amplification in a given patient. The strongest co-amplification pairs were ERBB2 and RARA (30 patients, RARA targeted by alitretinoin, respectively, ERBB2 i.a. by trastuzumab), followed by PIK3CA and SST (17 patients, PIK3CA targeted by copanlisib, respectively, SST by lanreotide)

To get more insight into genetic interactions in GC, particularly in terms of identifying multiple drug target CNV’s, we performed a network analysis focusing on genes that were co-amplified. Figure 2 shows the network of genes that were co-amplified in single patients (Supplementary Table 7). The most frequently co-amplified gene pairs were

- ERBB2 together with RARA in 30 of 393 cases
- PIK3CA with SST in 17 of 393 cases
- VGFRA with ERBB2 in 12 of 393 cases

### Drug response prediction: patient subgroup analysis

**Fig. 3:**
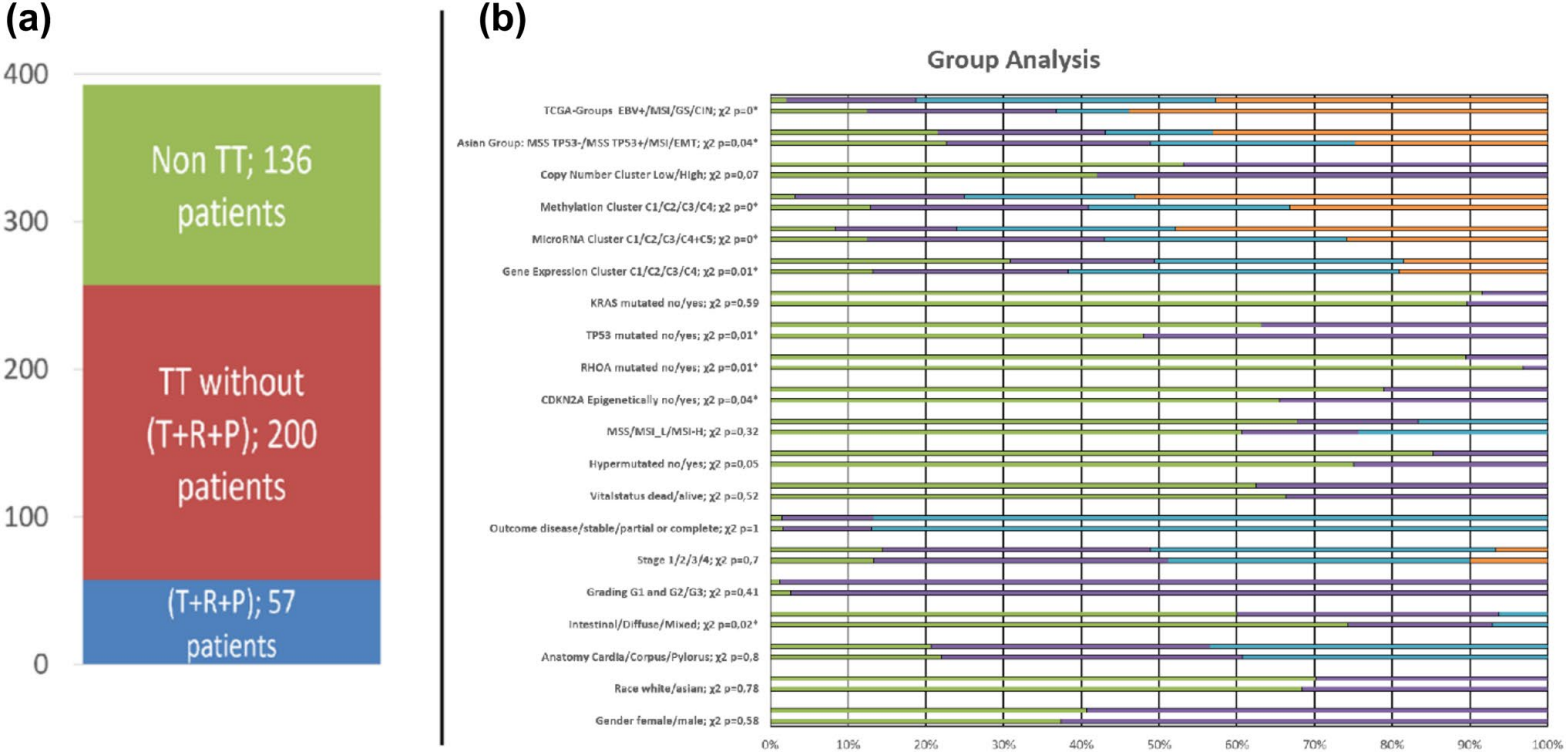
**a** The bar-chart represents the 393 TCGA patients analyzed that according to our in silico analysis are predicted to respond to approved target therapies (trastuzumab, ramucirumab, pembrolizumab (*T* + *R* + *P*) in blue); predicted to respond to other FDA-approved targeted drugs (red) and patients with no currently identifiable targeted option (green). **b** In every line, the upper bar corresponds to the patient group without therapeutically options, the lower bars (corresponding green bar in **a)** to those with therapeutically options (corresponding to red and blue bar in **a)**. Each bar is further stacked according to the description. From top to down, the following categories were included: STAD-TCGA classification, ACGR classification, CNV cluster, methylation cluster, microRNA cluster, gene expression cluster, KRAS mutated, TP53 mutated, RHOA mutated, CDK2NA epigenetically silenced, microsatellite instability, hypermutated, vital status, outcome, cancer staging, grading, Lauren classification, tumor location, race, gender. A *χ*^2^ analysis showed a difference between the patient groups with and without predicted options, whereby significant results are marked with a star (*, *p* < 0.05) (colour figure online)

Our analysis of targeted genes revealed that 65% of all patients show alterations in at least one gene with potential therapeutic options and 15% of all patients show alterations in ERBB2, PDCD1 or KDR, the gene targets of trastuzumab, pembrolizumab and ramucirumab, respectively. This means that a stunning 50% of patients with GC in the TCGA dataset might be treatable with alternative targeted drugs that have already been approved for use in patients with other cancer types (see Fig. 3a).

In order to test whether there is a specific subgroup of patients that would benefit from this analysis, we first identified patients with potentially targeted therapy options for GC for any FDA-approved targeted drugs and compared with patients with no targeted options for any FDA-approved cancer targeted drugs. We performed a classification of tissue samples based on criteria established by the molecular classification of the STAD-TCGA Consortium, clinical and pathological information, as well as the classification of the survival-based Asian Cancer Research Group (ACGR) into 20 subgroups (see Fig. 3b). We identified nine subgroups of patients that might particularly benefit from alternate targeted therapies (at a significance level of *p* < 0.05): (1) the STAD-TCGA Consortiums, the STAD-TCGA Consortiums clusters analysis of (2) copy number, (3) RNA, (4) miRNA and (5) methylation as well as (6) the ACGR Consortium, (7) Lauren classification and the mutation status of the genes (8) TP53 and (9) RHOA (see Fig. 3b and Supplementary Table 10).

### Drug response prediction: drug sensitivity versus drug resistance conferring mutations

We characterized FDA-approved drugs with respect to genetic alterations in GC patients that might confer resistance or sensitivity to the drug. Remarkably, in this analysis of the TCGA tissue set we found genetic modifications that are predicted to confer resistance to specific drugs (as defined by the curated databases) concurrent with alterations that would confer sensitivity to a drug. The resistance-associated genetic alterations would be expected to counteract sensitivity, for example in the case of cetuximab (Fig. 5). Therefore, due to mutations conferring resistance to drug treatment, our data indicate that cetuximab, as well as the absence of resistance mutation variants targeting specific by the second or third generation of EGFR TKIs (for example osimertinib, see Supplementary Table 13), cannot be considered to be effective in GC patients.

### Alternatives to trastuzumab in GC: bringing it all together

**Fig. 4:**
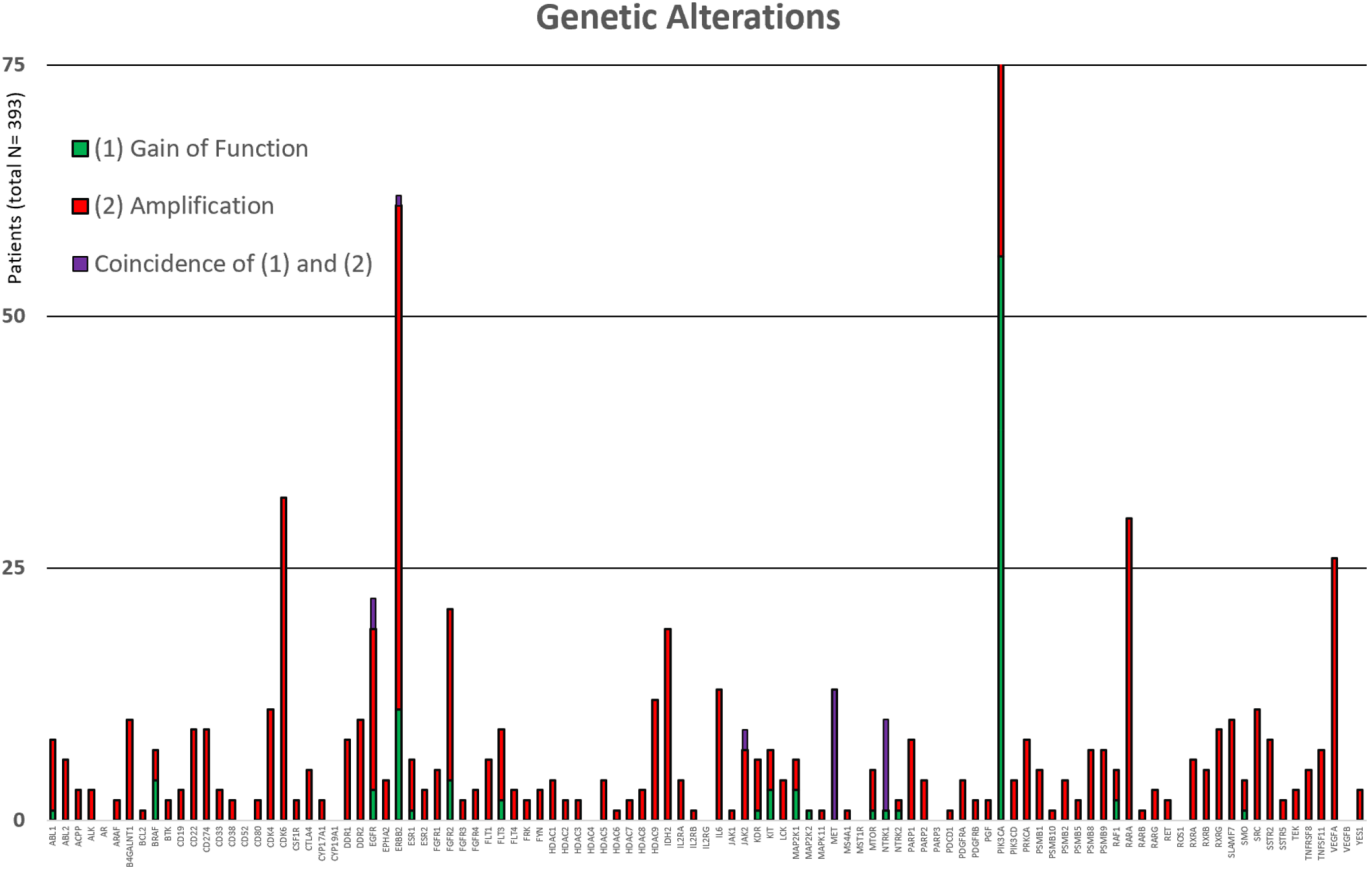
Listed is the number of patients (*y*-axis) in the gastric cancer TCGA cohort (total 393 patients) with either amplification (red), a gain of function (green) or a combination of mutations (purple) in a given gene (*x*-axis). Markedly PIK3CA has a high incidence of gain of function mutation (colour figure online)

**Fig. 5:**
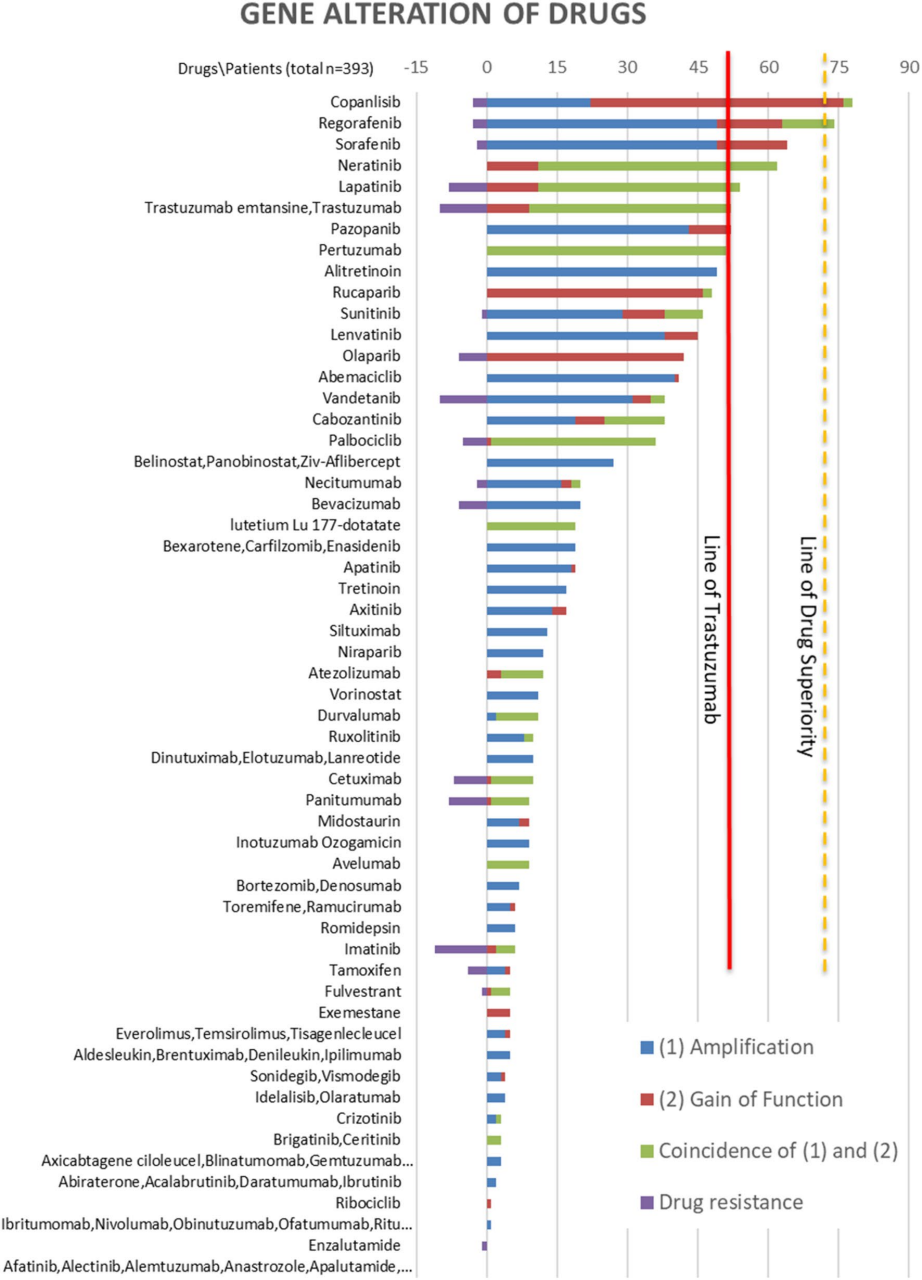
The *y*-axis lists all FDA-approved cancer drugs. The *x*-axis shows the number of GC patients with gene amplification (blue), gain of function mutation (red), both previous alterations (purple) and drug resistance (bright blue). Patients were only counted once within a given drug but could have alterations targeted by multiple drugs. The current standard of care drug trastuzumab is marked with a red line. The yellow line demarcates “significant drug response prediction” over trastuzumab as calculated in Table 1 (colour figure online)

In summary, Fig. 4 lists all gene alterations according to gain of function, CNV amplifications as well as their combinations (Supplementary Table 9). The gene with the highest number of alterations is PIK3CA (also see section on “mutation variants”). Of note is also the low occurrence of ramucirumab’s targeted protein encoding gene (KDR) in the TCGA patient dataset (Fig. 4). Moreover, Fig. 5 is an overview of the number of patients in the STAD-TCGA dataset that shows genetic alterations that would theoretically respond to approved targeted cancer therapy drugs. Trastuzumab targets Her2; therefore, other drugs targeting Her2-like lapatinib and pertuzumab show comparable results to trastuzumab. Surprisingly, our analysis shows that at least in the TCGA patient database there is an increased occurrence of alterations targeted by non-GC-approved drugs (namely copanlisib, regorafenib, sorafenib and neratinib) compared to alterations targeted by the GC-approved drug trastuzumab (Supplementary Tables 13 and 14). This suggests that more TCGA patients would be predicted to have responded to other FDA-approved drugs compared to the first in line therapy options.

To compare these subgroups of patients statistically, we counted the number of patients projected to respond to trastuzumab and selected drugs from our genetical drug response prediction analysis and compared the confidence intervals of the patient proportions (Table 1). Of note is that in the STAD-TCGA patient dataset, our drug response prediction suggests that an equal amount of patients would respond to sorafenib and trastuzumab, but significantly more patients would respond to copanlisib and regorafenib compared to trastuzumab. This identifies subgroups in GC cancer that could benefit from alternate therapy options. Importantly, sorafenib and regorafenib are currently in clinical trials [28–31].

**Table 1:** Prevalence and the confidence intervals for the difference between two proportions of patient populations for several drugs compared to trastuzumab (52 patients with genetically predicted drug response out of total 393 patients). In addition, the lower row lists clinical trials in respect to the respective drugs for GC

	Trastuzumab	Copanlisib	Regorafenib	Sorafenib
Prevalence (Patients with genetically predicted drug response out of total patients)	13% (52/393)	20% (78/393)	19% (74/393)	16% (64/393)
CI drug versus trastuzumab		(0.01–0.12)	(0.01–0.11)	(− 0.02 to 0.08)
Trials	ToGA trial [4] OS: 13.8 versus 11.1, HR: 0.74 *p* = 0.0046	No trial	INTEGRATE trial [35] OS: 5.8 versus 4.5, HR 0.74 *p* = 0.147	Multicenter phase II study [28]. Neutropenia (9.8%), thrombocytopenia (7.3%), neurotoxicity (4.9%), diarrhea (4.9%)

## Discussion

Our data clearly confirm previous observations that GC is linked to a high incidence of alterations of ERBB2 encoding the HER2 receptor proto-oncogene [3] and confirm the viability of trastuzumab as a therapy option for GC. Further, other drugs which interact with the HER2 signaling pathway could theoretically be comparable therapy options, e.g., lapatinib and pertuzumab [32, 33]. According to our in silico analysis, currently approved targeted therapy drugs for GC (trastuzumab, pembrolizumab and ramucirumab) are predicted to benefit up to 15% of GC TCGA patients. In contrast, an astonishing 50% of the patients would be predicted to benefit from already-FDA-approved alternative targeted therapies. This result underlines the heterogenous genetic basis for GC in the TCGA dataset which has also been described in other GC samples [1, 34, 35].

A major GC target identified in this analysis is the high incidence of PIK3CA mutation variants. Here, we found two main hot spots for activating mutations, the 542/545 region of the helical domain and the 1047 region of the kinase domain. PIK3CA, and its interaction with the AKT and mTOR pathways, has been subject of recent research and development. Specifically PI3K inhibition might be of limited success in recent clinical trials for other cancer types but has not yet been analyzed for GC [36, 37]. A PI3K inhibitor—copanlisib that preferentially acts on PIK3 alpha and delta isoforms—has recently been approved by the FDA for follicular lymphoma [38]. Among the TCGA patients for GC, we find that copanlisib shows promise as an alternative to trastuzumab, suggesting that testing for tumors/patients with PIK3CA mutations in GC followed by trials with copanlisib could be promising.

Other drugs identified here for therapy options are sorafenib with 16% of the TCGA GC patients (64/total 393 patients), cobozantinib (20%) and regorafenib (19%), which showed a relatively good effectiveness both from our in silico analysis outlined in Fig. 5. These targeted drugs are characterized by lacking specificity for their targets, again arguing for a quite diffuse nature of GC. Sorafenib, an inhibitor for multiple kinases targeting VEGFR-2, VEGFR-3 and PDGFR in combination with oxalipatin chemotherapy, was analyzed in a multicenter phase II study in advanced GC. The trial showed a high incidence of grade 3–4 toxic effects: neutropenia (9.8%), thrombocytopenia (7.3%), neurotoxicity (4.9%) and diarrhea (4.9%) [28], which makes it difficult to draw clear conclusions.

Regorafenib is another oral multikinase inhibitor which stimulates protein kinases including VEGFR and RAF, demonstrating an efficacy in numerous cancers in clinical trials. In 2012, regorafenib has received the FDA approval for treatment of the advanced colorectal cancer (CORRECT trial [29]) and in 2013 also for advanced gastrointestinal stromal tumors (GRID trial [30]). In a phase II placebo-controlled double-blinded trial, regorafenib has been investigated for efficacy in the treatment of refractory advanced gastric cancer, and a significantly longer median PFS for the regorafenib group versus placebo was noted (regorafenib, 2.6 months; 95% CI, 1.8–3.1 and placebo, 0.9 months; 95% CI, 0.9–0.9; hazard ratio [HR], 0.40; 95% CI, 0.28–0.59; *p* < .001, INTEGRATE trial [31]). Toxicity rates of serious adverse events were 32% versus 18% in patients treated on the experimental and control arms, respectively. Based on the good overall survival results, regorafenib is further being assessed in an ongoing randomized phase III trial (INTEGRATE II). Both sorafenib and regorafenib were identified in our analysis as potential candidates and have already undergone testing in GC with promising results in case of the latter. No clinical trials have been published for cabozantinib in GC to date, but our study indicates that this would be another interesting candidate.

Overall, it must be re-iterated that our study is on the basis of theoretical considerations and analysis of the different databases as well as current knowledge on genes and drug targets. Clinical studies are needed to confirm our theoretical results.

Another finding was the low presence of the alterations targeted by ramucirumab which would predict that of the patients sampled in the TCGA dataset only few would be predicted to respond. This suggests that the relatively marginal statistical effectiveness observed in the REGARD trial [6] may not be surprising for an unselected population (median OS: 5.2 vs. 3.8 months; HR 0.776; 95% CI: 0.603–0.998; *p* = 0.047) [6]. A selected population may have led to a different effectiveness. It follows that patient selection based on molecular profiling is pivotal and should be at the core for future targeted therapy choices, hopefully increasing the success rate of GC therapy. Lastly, our in silico analysis showed that targeting EGFR in GC could be problematic due to the high degree mutations predicted to confer drug resistance (e.g., cetuximab) as well as the absence of resistance mutation variants targeted specifically by the second or third generation of EGFR TKIs (e.g., Osimertinib targets EGFR T790M).

Of note, however, is that the data are biased toward the TCGA dataset. These samples were collected predominantly from patients treated with curative intent and earlier stages of cancer as well as prior to chemotherapy and radiation. Disease progression—especially during treatment—is often also accompanied by genomic evolution and progression of clonal cancer subpopulations. However, our drug response prediction is agnostic to the stage of cancer but rather aims at the underlying genetic basis and at the initiating and pushing driver mutations of the disease. Consequently, as patients show progression, we hypothesize that the progressing tumor tissue and provided medication require re-evaluation.

Further, we have considered only single-agent therapies in this analysis. Recent studies have also highlighted the benefit of using multiple drug targets [39, 40], and indeed, our dataset also shows co-amplification pairs (Fig. 2) and many patients with multiple druggable targets (supp. Table 15). As studies using multiple drugs progress and are approved, these will need to be integrated into our drug response prediction algorithm.

Clinical studies to validate the drug response prediction presented here are urgently needed. We believe that this approach is very promising as it has been shown before that biomarker-driven trials have better outcomes than trials lacking biomarkers [41]. Further, the recent WINTHER trial for advanced cancer patients showed that a higher matching score of genomic alterations to medication correlated with longer progression-free survival and even overall survival especially for patients with an ECOG performance status of 0 [42]. We consequently believe in the usefulness of cancer genetics to drive therapy recommendations and improve clinical outcome.

It is remarkable that all targeted drugs used so far for GC treatment show only a relatively minor improvement of survival rate by 2–3 month compared to breakthroughs in other types of cancer-like imatinib for chronic myeloid leukemia, which results in a 82% 10-year overall survival as shown in the study by Cohen et al. [43] as well as in Hehlmann et al. [44]. Our study clearly argues that GC consists of subgroups which should themselves be amenable to specific treatment options. We argue for a strategy of personalized treatment of GC patients with an expanded mutational analysis panel, combined with an unbiased sequencing option. This would need to be followed both by careful analysis for genetic alterations treatable with current FDA-approved targeted drugs as well as resistance conferring alterations. The VIKTORY trial showed such an approach. It used an umbrella platform trial design with preplanned molecular profiling to assign patients with advanced gastric cancer to molecularly matched therapies [45].

As far as biased options go, this in silico approach highlights several interesting genes which were significantly increased in some TCGA GC cases and might present good targets for therapy. As the prices for unbiased approaches come down, true personalized medicine should become a feasible option for all cancer types.

## Electronic supplementary material

Below is the link to the electronic supplementary material.

Supplementary material 1 (XLSX 9142 kb)
